# Supplementary material for: Adherence to the EAT-Lancet reference diet is associated with a reduced risk of incident cancer and all-cause mortality in UK adults
Source: One Earth. 2023 Dec 15;6(12):1726–34. doi: 10.1016/j.oneear.2023.11.002 (PMC10731983; doi:10.1016/j.oneear.2023.11.002)
Supplement: Document S1. Figure S1, Table S1 and Note S1 [file mmc1.pdf]

One Earth, Volume *6*

Supplemental information

Adherence to the EAT-Lancet reference diet is associated with a reduced risk of incident cancer and all-cause mortality in UK adults

Nena Karavasiloglou, Alysha S. Thompson, Giulia Pestoni, Anika Knuppel, Keren Papier, Aedín Cassidy, Tilman Kühn, and Sabine Rohrmann

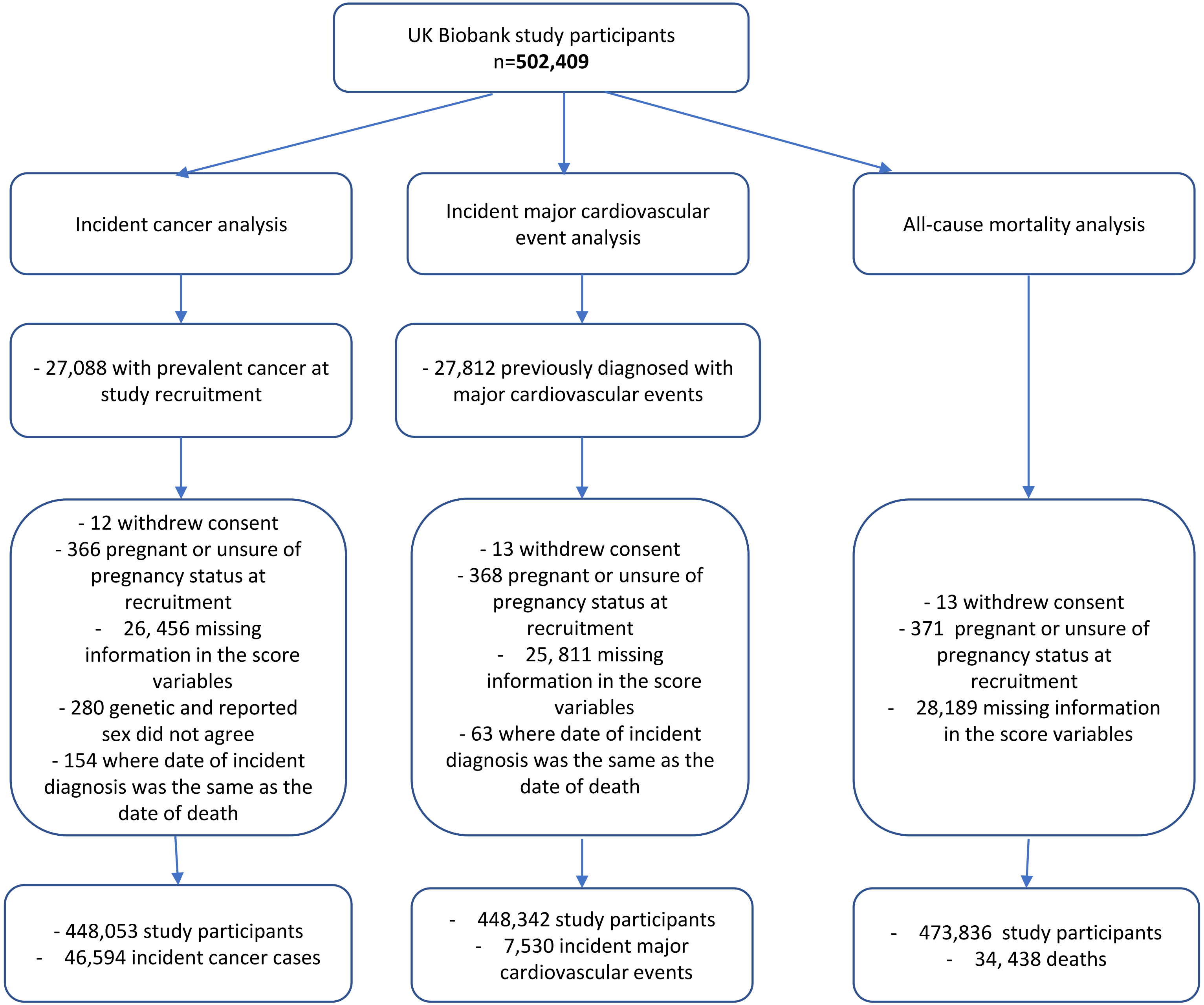

Figure S1. Flowchart of study population

Table S1. Operationalization of the components of the EAT-Lancet reference diet using the UK Biobank Touchscreen questionnaire [a,b]

EAT-Lancet reference diet component	Food groups in the UK Biobank	Cut-offs of daily dietary intake (males/females)
Grains (incl. rice, wheat, corn, and other)	Partial component, estimated based on bread intake and cereal intake and on the question "Which of the following do you never eat?", with answer wheat products	Sex-specific cohort-based median of consumers.<br>> 91g/ 70g (median): 0<br>≤ median: 1<br>Participants reporting avoidance of wheat products and reported no consumption of cereals received the one point.
Vegetables	Cooked vegetable and salad / raw vegetable intake	200-600g/160-480g
Fruits	Fresh fruit and dried fruit intake	100-300g/80-240g
Dairy foods (incl. whole milk or equivalents)	Partial component, estimated based on cheese intake and on the question "Which of the following do you never eat?", with possible answer dairy products	Sex-specific cohort-based median of consumers.<br>> 13g/ 13g (median): 0<br>≤ median: 1<br><br>Participants reporting avoidance of dairy received one point
Protein sources		
Beef, lamb, and pork	Beef, lamb/mutton intake and pork intake	≤28g/22g
Chicken and other poultry	Poultry	≤58g/46g
Eggs	Binary outcome, based on the question "Which of the following do you never eat?", with possible answer eggs	Participants avoiding eggs received one point
Fish	Oily fish and non-oily fish intake	≤100g/80g
Added fats		
Unsaturated oils	Binary outcome, based on spread type and non-butter spread type details	Those not using spreads or using Benecol received the full point. The rest and those using hard margarine received one point
Saturated oils	Binary outcome, based on spread type and non-butter spread type details	Those not using spreads or using Benecol received the full point. The rest and those using hard margarine received one point
Added sugars (incl. all sugars)	Binary outcome, based on the question "Which of the following do you never eat?", with possible answer sugar or foods/ drinks containing sugar	Participants avoiding sugar received one point

[a] The EAT-Lancet reference diet cut-offs are based on the report of the EAT-Lancet Commission, to reflect a diet of 2,500 kcal/day. (Summary Report of the EAT-Lancet Commission. Food Planet Health. Healthy Diets From Sustainable Food Systems, 2019). Our score is based, to the extend possible, on the work by Knuppel et al. 2019. Consumption within each component's cut-offs results in one point being awarded, otherwise, zero points are awarded. When the information in the UK Biobank Touchscreen questionnaire was not detailed enough to allow us to follow the score by Knuppel et al. 2019, the sex-specific cohort medians were used as a cut-off.

[b] The dietary intake for females was rescaled to reflect a diet of 2,000 kcal/day, rounded to the nearest whole number. Due to the selected food groups in the Touchscreen questionnaire, it was not possible to estimate the consumption of tuber or starchy vegetables, legumes, and nuts, and include them in the score.

Note S1. STROBE Statement for cohort studies

	Item No	Recommendation	Page No
**Title and abstract**	1	(*a*) Indicate the study's design with a commonly used term in the title or the abstract	2
		(*b*) Provide in the abstract an informative and balanced summary of what was done and what was found	2
**Introduction**			
Background/rationale	2	Explain the scientific background and rationale for the investigation being reported	3-4
Objectives	3	State specific objectives, including any prespecified hypotheses	4
**Methods**			
Study design	4	Present key elements of study design early in the paper	4-8
Setting	5	Describe the setting, locations, and relevant dates, including periods of recruitment, exposure, follow-up, and data collection	4-8
Participants	6	(*a*) Give the eligibility criteria, and the sources and methods of selection of participants. Describe methods of follow-up	4, 6
		(*b*) For matched studies, give matching criteria and number of exposed and unexposed	
Variables	7	Clearly define all outcomes, exposures, predictors, potential confounders, and effect modifiers. Give diagnostic criteria, if applicable	4-8
Data sources/ measurement	8*	For each variable of interest, give sources of data and details of methods of assessment (measurement). Describe comparability of assessment methods if there is more than one group	4-8
Bias	9	Describe any efforts to address potential sources of bias	8
Study size	10	Explain how the study size was arrived at	4, Fig1
Quantitative variables	11	Explain how quantitative variables were handled in the analyses. If applicable, describe which groupings were chosen and why	5-8
Statistical methods	12	(*a*) Describe all statistical methods, including those used to control for confounding	7-8
		(*b*) Describe any methods used to examine subgroups and interactions	8
		(*c*) Explain how missing data were addressed	7-8
		(*d*) If applicable, explain how loss to follow-up was addressed	
		(*e*) Describe any sensitivity analyses	8
**Results**			
Participants	13*	(a) Report numbers of individuals at each stage of study—eg numbers potentially eligible, examined for eligibility, confirmed eligible, included in the study, completing follow-up, and analysed	Fig1
		(b) Give reasons for non-participation at each stage	Fig1
		(c) Consider use of a flow diagram	Fig1
Descriptive data	14*	(a) Give characteristics of study participants (eg demographic, clinical, social) and information on exposures and potential confounders	Table 2
		(b) Indicate number of participants with missing data for each variable of interest	Table 2
		(c) Summarise follow-up time (eg, average and total amount)	8

Outcome data	15*	Report numbers of outcome events or summary measures over time	Tables 3-5
Main results	16	(*a*) Give unadjusted estimates and, if applicable, confounder-adjusted estimates and their precision (eg, 95% confidence interval). Make clear which confounders were adjusted for and why they were included	Tables 3-5
		(*b*) Report category boundaries when continuous variables were categorized	Tables 3-5
		(*c*) If relevant, consider translating estimates of relative risk into absolute risk for a meaningful time period	n/a
Other analyses	17	Report other analyses done—eg analyses of subgroups and interactions, and sensitivity analyses	8, Fig 2-4
**Discussion**			
Key results	18	Summarise key results with reference to study objectives	9-10
Limitations	19	Discuss limitations of the study, taking into account sources of potential bias or imprecision. Discuss both direction and magnitude of any potential bias	10-11
Interpretation	20	Give a cautious overall interpretation of results considering objectives, limitations, multiplicity of analyses, results from similar studies, and other relevant evidence	11
Generalisability	21	Discuss the generalisability (external validity) of the study results	11
**Other information**			
Funding	22	Give the source of funding and the role of the funders for the present study and, if applicable, for the original study on which the present article is based	12

*Give information separately for exposed and unexposed groups.

Note: An Explanation and Elaboration article discusses each checklist item and gives methodological background and published examples of transparent reporting. The STROBE checklist is best used in conjunction with this article (freely available on the Web sites of PLoS Medicine at http://www.plosmedicine.org/, Annals of Internal Medicine at http://www.annals.org/, and Epidemiology at http://www.epidem.com/). Information on the STROBE Initiative is available at http://www.strobe-statement.org.

Supplemental reference list

EAT-Lancet Commission. Summary Report of the EAT-Lancet Commission. Food Planet Health. Healthy Diets From Sustainable Food Systems. 2019.

Knuppel A, Papier K, Key TJ, Travis RC. EAT-Lancet score and major health outcomes: the EPIC-Oxford study. *The Lancet* 2019; **394**: 213–4.
