# Supplementary material for: Adherence to the EAT-Lancet reference diet is associated with a reduced risk of incident cancer and all-cause mortality in UK adults
Source: One Earth. 2023 Dec 15;6(12):1726–34. doi: 10.1016/j.oneear.2023.11.002 (PMC10731983; doi:10.1016/j.oneear.2023.11.002)
Supplement: Document S2. Article plus supplemental information [file mmc2.pdf]

One Earth

Article

Adherence to the EAT-Lancet reference diet is associated with a reduced risk of incident cancer and all-cause mortality in UK adults

Graphical abstract

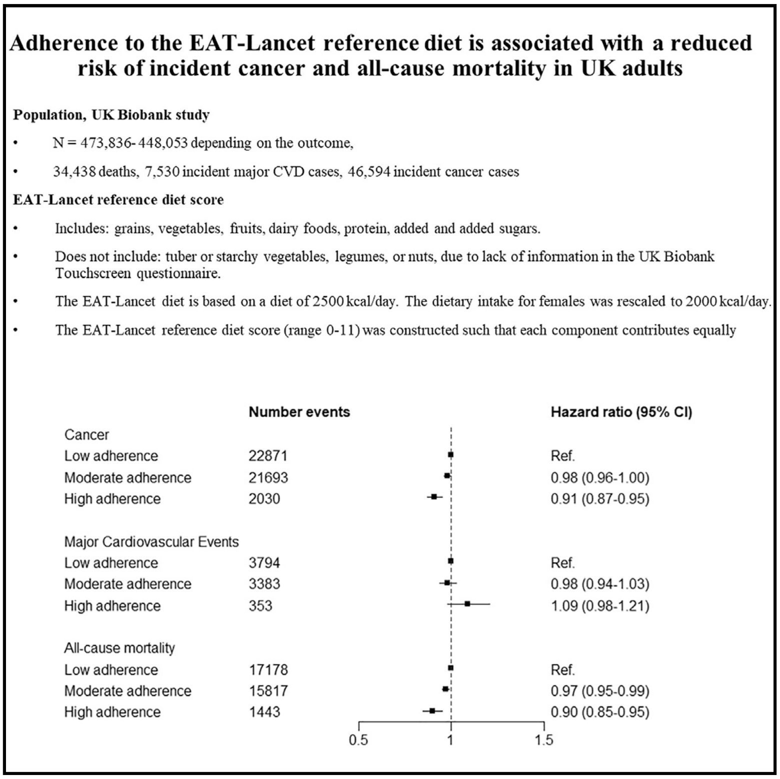

Highlights

- Adherence to the EAT-Lancet reference diet was associated with lower cancer risk
- Adherence to the reference diet was inversely associated with all-cause mortality
- No association between the reference diet and cardiovascular diseases was observed

Authors

Nena Karavasiloglou, Alysha S. Thompson, Giulia Pestoni, ..., Aedín Cassidy, Tilman Kühn, Sabine Rohrmann

Correspondence

sabine.rohrmann@uzh.ch

In brief

Food systems are significant contributors to the global environmental emergency. Therefore, we investigated the association between the EAT-Lancet reference diet, a diet within the planetary boundaries, and incident cancer, incident major cardiovascular events, and all-cause mortality. People who closely followed the planetary health diet had a lower risk for cancer and mortality from all causes than those who did not closely follow the planetary health diet. Hence, following the EAT-Lancet reference diet could have benefits for non-communicable disease prevention.

Karavasiloglou et al., 2023, One Earth *6*, 1726–1734
December 15, 2023 © 2023 The Authors. Published by Elsevier Inc.
https://doi.org/10.1016/j.oneear.2023.11.002

CellPress

CellPress OPEN ACCESS

One Earth

Article

Adherence to the EAT-Lancet reference diet is associated with a reduced risk of incident cancer and all-cause mortality in UK adults

Nena Karavasiloglou,[1,2,3] Alysha S. Thompson,[4] Giulia Pestoni,[1,5] Anika Knuppel,[6] Keren Papier,[7] Aedín Cassidy,[4] Tilman Kühn,[4,8] and Sabine Rohrmann[1,2,9,*]

[1]Division of Chronic Disease Epidemiology, Epidemiology, Biostatistics and Prevention Institute (EBPI), University of Zurich, Zurich, Switzerland
[2]Cancer Registry of the Cantons of Zurich, Zug, Schaffhausen, and Schwyz, University Hospital Zurich, Zurich, Switzerland
[3]European Food Safety Authority, Parma, Italy
[4]The Institute for Global Food Security, School of Biological Sciences, Queen's University Belfast, Belfast, Northern Ireland, UK
[5]Nutrition Group, Health Department, Swiss Distance University of Applied Sciences, Zurich, Switzerland
[6]MRC Unit for Lifelong Health and Ageing at UCL, Institute of Cardiovascular Science, University College London, London, UK
[7]Cancer Epidemiology Unit, Nuffield Department of Population Health, University of Oxford, Oxford, UK
[8]Heidelberg Institute of Global Health (HIGH), Faculty of Medicine and University Hospital, Heidelberg, Germany
[9]Lead contact
*Correspondence: sabine.rohrmann@uzh.ch
https://doi.org/10.1016/j.oneear.2023.11.002

SCIENCE FOR SOCIETY There is an increasing interest in plant-based diets (i.e., diets rich in plant-based products that include little, if any, animal products), due to their environmental sustainability and potential health benefits. Recently, international experts recommended a mostly plant-based, sustainable diet referred to as the "planetary health diet" We investigated to find out if individuals adhering to the planetary health diet proposed by the EAT-Lancet Commission are at a lower risk for cancer, cardiovascular disease, or mortality from all causes. In our study, people who closely followed the planetary health diet had a lower risk for cancer and mortality from all causes than those who did not closely follow the planetary health diet. Our study adds to the existing literature that following the EAT-Lancet reference diet could have benefits for non-communicable disease prevention.

SUMMARY

Food systems have been identified as significant contributors to the global environmental emergency. However, there is no universally agreed-upon definition of what constitutes a planetary healthy, sustainable diet. In our study, we investigated the association between the EAT-Lancet reference diet, a diet within the planetary boundaries, and incident cancer, incident major cardiovascular events, and all-cause mortality. Higher adherence to the EAT-Lancet reference diet was associated with lower incident cancer risk (hazard ratio $[HR]_{continuous}$: 0.99; 95% confidence interval [CI]: 0.98–0.99]) and lower all-cause mortality ($HR_{continuous}$: 0.98; 95% CI: 0.98–0.99), while mostly null associations were detected for major cardiovascular event risk ($HR_{continuous}$: 1.00; 95% CI: 0.98–1.01). Stratified analyses using potentially modifiable risk factors led to similar results. Our findings, in conjunction with the existing literature, support that adoption of the EAT-Lancet reference diet could have a benefit for the prevention of non-communicable diseases.

INTRODUCTION

Food systems significantly contribute to the global environmental emergency, with approximately one-third of anthropogenic greenhouse gas emissions being attributed to them,[1] but there is no universally agreed-upon definition of what constitutes a planetary healthy, sustainable diet. While the Food and Agriculture Organization (FAO)[2] has recently proposed a definition of sustainable diets, most scientists have long considered dietary patterns promoting a high proportion of plant-based foods in the diet as sustainable. The common characteristic of all these plant-based dietary patterns is the reduced consumption or

 © 2023 The Authors. Published by Elsevier Inc.
This is an open access article under the CC BY license (http://creativecommons.org/licenses/by/4.0/).

entire exclusion of animal products from the diet. However, these patterns include a diverse range of products (both healthy and unhealthy), resulting in intakes of different diet quality.

In 2019, the EAT-Lancet Commission on Food, Planet, Health proposed the "healthy reference diet," also referred to as the EAT-Lancet reference diet, a mainly plant-based diet that, according to the EAT-Lancet Commission, reflects a sustainable food system.[3,4] The EAT-Lancet reference diet is close to most national food-based dietary or disease prevention (e.g., cancer prevention) guidelines but takes a much stricter approach regarding animal-based product consumption, in particular, meat and dairy products. According to the EAT-Lancet Commission, the reference diet addresses the diet and farming aspects of environmental changes better than most national food-based dietary guidelines.[4]

According to the Global Burden of Disease Study, cardiovascular disease was the leading cause of diet-related deaths globally in 2017, followed by cancer.[5] Dietary factors have been associated with incident cardiovascular disease[6] and incident cancer risk,[7–11] as well as all-cause mortality.[12] Studies suggest that 5%–20% of incident cardiovascular disease cases, incident cancer cases, or deaths are attributable to dietary factors[13,14] (the exact proportion depends on the dietary factor, the study population, and the investigated endpoint). Furthermore, the existing evidence suggests that dietary patterns with high consumption of plant-based foods are associated with a lower risk of many non-communicable diseases.[6,15,16]

Despite widespread media publicity and interest in the scientific community, few studies have investigated if the EAT-Lancet reference diet is also beneficial for human health using large population-based data.[17–24] Most reported an inverse association between the EAT-Lancet reference diet and their outcome of interest, despite the different methodologies they used (e.g., assessment of dietary intake, operationalization of the EAT-Lancet reference diet). To date, only one study has investigated the association between the EAT-Lancet reference diet and cancer in approximately 60,000 participants over 8 years of follow-up and reported an overall null association.[18]

In our study, we investigated using the largest sample size to date, the association between the EAT-Lancet reference diet and incident cancer, incident major cardiovascular events, and all-cause mortality, overall and by potential effect modifiers, in the UK Biobank cohort. Our findings showed an inverse association between higher adherence to the EAT-Lancet reference diet and cancer risk and all-cause mortality. Our study provides evidence suggesting that adherence to the diet proposed by the EAT-Lancet expert commission could not only aid in the mitigation of the climate emergency but could also be beneficial for the reduction of non-communicable diseases and all-cause mortality.

RESULTS

Baseline study population characteristics

After an average follow-up of 11.5 years (mean: 10.49 years for cancer, 11.98 years for major cardiovascular events, 11.98 years for mortality), 46,594 incident cancer cases and 7,530 incident major cardiovascular events were diagnosed in the UK Biobank cohort, while 34,438 people died.

Participants with higher EAT-Lancet reference diet scores (i.e., higher adherence to the diet proposed by the EAT-Lancet Commission; Table 1) were more likely to be female, more physically active, and to have obtained college/university degree, while they were less likely to report being current smokers or drink alcohol.

EAT-Lancet diet and non-communicable disease risk

Higher adherence to the EAT-Lancet reference diet was associated with lower cancer risk (hazard ratio $[HR]_{continuous}$: 0.99; 95% confidence interval [CI]: 0.98–0.99, per one-unit increase in the score; $HR_{low\ vs.\ high}$: 0.91; 95% CI: 0.87–0.95; Table 2). In selected cancer types, higher adherence of the EAT-Lancet reference diet was not associated with breast ($HR_{continuous}$: 0.99; 95% CI: 0.98–1.00; $HR_{low\ vs.\ high}$: 0.94; 95% CI: 0.85–1.04), colorectal ($HR_{continuous}$: 0.98; 95% CI: 0.98–1.00; $HR_{low\ vs.\ high}$: 0.90; 0.78–1.03), or prostate ($HR_{continuous}$: 0.99; 95% CI: 0.98–1.01; $HR_{low\ vs.\ high}$: 0.93; 95% CI: 0.84–1.04) cancer risk. Additional adjustment for reproductive factors (for breast cancer) or first-degree family history and previous cancer screening did not substantially affect the cancer-site specific results.

Higher adherence to the EAT-Lancet reference diet was not associated with a lower risk for major cardiovascular events ($HR_{continuous}$: 1.00; 95% CI: 0.98–1.01; $HR_{low\ vs.\ high}$: 1.09; 95% CI: 0.98–1.21: Table 3). Similar null associations were seen in analyses on total stroke ($HR_{continuous}$: 0.99; 95% CI: 0.97–1.02; $HR_{low\ vs.\ high}$: 1.04; 95% CI: 0.87–1.24), the main subtypes of stroke, or myocardial infarction ($HR_{continuous}$: 1.00; 95% CI: 0.99–1.02; $HR_{low\ vs.\ high}$: 1.12; 95% CI: 0.98–1.29). Upon additional adjustment for first-degree family history of cardiovascular disease, the overall association became inverse ($HR_{continuous}$: 0.97; 95% CI: 0.96–0.98; $HR_{low\ vs.\ high}$: 0.94; 95% CI: 0.84–1.05), while the total stroke and myocardial infarction analyses did not change substantially.

Higher adherence to the EAT-Lancet reference diet was associated with lower mortality risk ($HR_{continuous}$: 0.98; 95% CI: 0.98–0.99; $HR_{low\ vs.\ high}$: 0.90; 95% CI: 0.85–0.95; Table 4).

Stratified analysis

The results of stratified analysis are shown in Figure 1A (cancer incidence), Figure 1B (incident major cardiovascular events), and Figure 1C (mortality). There were no substantial differences observed between the different groups for cancer risk, major cardiovascular event risk, or all-cause mortality. The association between the EAT-Lancet reference diet and cancer was statistically significant for males, but not females. Restricting the analyses to study participants with at least 2 years of follow-up time did not alter the interpretation of the results (Figure 1).

DISCUSSION

In the largest study to date on the link between the EAT-Lancet reference diet and non-communicable diseases, we observed inverse associations between the EAT-Lancet reference diet and risk of all-cause mortality and incident cancer.

Despite the widespread media exposure of the diet proposed by the EAT-Lancet Commission, few studies thus far have investigated its association with non-communicable diseases. To the best of our knowledge, only one observational study exists on

Table 1. Baseline characteristics of the study population, overall and by categories reflecting adherence to the EAT-Lancet reference diet

	EAT-Lancet reference diet score			
	Total (n = 473,836)	Low adherence, 0–4 points (n = 229,968)	Moderate adherence, 5–7 points (n = 221,727)	High adherence, 8–11 points (n = 22,141)
Age at recruitment, mean (SD)	56.50 (8.08)	56.33 (8.15)	56.64 (8.02)	57.32 (7.73)
Sex - Female, %	54.52	50.95	57.15	65.23
Highest level of attained education, %				
None of the following	16.36	15.38	17.17	18.36
CSEs/O-levels/GCSEs[a], or equivalent	25.55	25.89	25.34	24.05
NVQ/HND/HNC/A-levels/AS-levels or equivalent	17.19	17.38	17.04	16.74
Other professional qualifications	28.45	29.40	27.66	26.62
College/university degree	11.55	11.20	11.77	12.95
Prefer not to answer/Missing	0.90	0.75	1.02	1.29
Smoking status, %				
Never smokers	54.63	54.35	54.84	55.37
Former smokers	34.67	33.96	35.21	36.70
Current smokers	10.39	11.42	9.60	7.52
Prefer not to answer/Missing	0.32	0.27	0.35	0.42
Body mass index, %				
$\leq$18.5 kg/m^2	0.50	0.55	0.45	0.53
18.5–24.9 kg/m^2	32.48	33.04	31.94	32.04
25.0–29.9 kg/m^2	42.37	41.83	42.87	43.04
$\geq$30 kg/m^2	24.15	24.15	24.19	23.78
Missing	0.49	0.42	0.55	0.60
Alcohol intake status, %				
Never drinkers	4.15	3.40	4.68	6.59
Former drinkers	3.43	3.12	3.65	4.58
Current drinkers	92.34	93.42	91.59	88.76
Prefer not to answer/Missing	0.08	0.07	0.09	0.07
Physical activity, %				
Less than 75 min per week	31.85	32.37	31.58	29.19
75–150 min per week	13.30	13.33	13.31	12.86
150 min or more per week	49.47	49.16	49.48	52.52
Prefer not to answer/Missing	5.38	5.14	5.63	5.43

[a]A-level: General Certificate of Education Advanced level; AS-levels: General Certificate of Education Advanced Supplementary level; CSE: Certificate of Secondary Education; HNC: Higher National Certificate; HND: Higher National Diploma; GCSE: General Certificate of Secondary Education; NVQ: National Vocational Qualification; O-level: General Certificate of Education Ordinary level; SD: Standard deviation.

the association between the EAT-Lancet reference diet and cancer risk. Berthy et al., using data from the NutriNet-Santé cohort, reported a null association overall. An inverse association was seen for specific population groups (e.g., females) without adjustment for body mass index (BMI).[18] These results are in slight contrast to our results, where we found that higher adherence was associated with lower risk for cancer. In our population, a statistically significant inverse association between the EAT-Lancet reference diet and cancer risk was observed for male participants in the fully adjusted model.

Studies investigating the association between the EAT-Lancet reference diet and cardiovascular diseases have reported inverse or null associations. Higher adherence to the EAT-Lancet reference diet has been inversely associated with lower risk of heart disease,[17,21,23] diabetes,[17,24] and stroke[19] in the literature, but null associations have been reported in some studies for these endpoints.[17,18,20] These inconsistencies in the findings could potentially be attributed to differences in the underlying structure of the cohorts, the way the score was constructed, the length of the follow-up, as well as analytical decisions, or they could in fact reflect differences in the true association. For all-cause mortality, an inverse association has been reported,[21,22] but not in all[17] studies.

In the absence of a standardized, universally accepted scoring system, it is inevitable that different groups will interpret and operationalize the EAT-Lancet reference diet differently. Despite

Table 2. The association between the EAT-Lancet reference diet score and incident cancer risk

	All incident cancer diagnoses HR (95% CI)			Breast cancer HR (95% CI)					Colorectal cancer HR (95% CI)					Prostate cancer HR (95% CI)				
EAT-Lancet reference diet score	Number of cases	Model 1 [a]	Model 2 [b]	Number of cases	Model 1 [a]	Model 2 [b]	Model 3a [c]	Model 3b [d]	Number of cases	Model 1 [a]	Model 2 [b]	Model 3a	Model 3b [d]	Number of cases	Model 1 [a]	Model 2 [b]	Model 3a	Model 3b [d]
Continuous, 0-11	46,594	0.99 (0.98–0.99)	0.99 (0.98–0.99)	8,516	0.99 (0.98–1.00)	0.99 (0.98–1.00)	0.99 (0.98–1.00)	0.99 (0.98–1.00)	5,120	0.98 (0.96–1.00)	0.98 (0.96–1.00)	–	0.98 (0.97–1.00)	10,150	0.99 (0.98–1.01)	0.99 (0.98–1.01)	–	0.99 (0.98–1.00)
Low adherence, 0-4	22,871	Ref.	Ref.	3,919	Ref.	Ref.	Ref.	Ref.	2,508	Ref.	Ref.	–	Ref.	5,173	Ref.	Ref.	–	Ref.
Moderate adherence, 5-7	21,693	0.98 (0.96–1.00)	0.98 (0.96–1.00)	4,140	0.98 (0.93–1.02)	0.98 (0.94–1.02)	0.98 (0.93–1.02)	0.98 (0.93–1.02)	2,396	1.00 (0.94–1.05)	1.00 (0.94–1.05)	–	1.00 (0.94–1.05)	4,595	1.00 (0.96–1.04)	1.00 (0.96–1.04)	–	0.99 (0.95–1.03)
High adherence, 8-11	2,030	0.90 (0.86–0.94)	0.91 (0.87–0.95)	457	0.93 (0.85–1.03)	0.94 (0.85–1.04)	0.94 (0.85–1.03)	0.94 (0.85–1.04)	216	0.90 (0.78–1.03)	0.90 (0.78–1.03)	–	0.90 (0.78–1.03)	382	0.94 (0.85–1.05)	0.94 (0.85–1.04)	–	0.93 (0.84–1.03)

Abbreviations: CI: confidence interval; HR: hazard ratio. In analyses with breast and prostate cancer as outcomes, only participants with reported sex as female and male, respectively, were included.

[a]Model based on age, sex, and region.

[b]Model based on age, sex, and region plus further adjustment for smoking status, body mass index, physical activity, highest level of attained education, Townsend deprivation index, and alcohol intake status.

[c]Model based on age, sex, region, smoking status, body mass index, physical activity, highest level of attained education, Townsend deprivation index, and alcohol intake status (Model 2), further adjusted for reproductive factors.

[d]Model based on age, sex, region, smoking status, body mass index, physical activity, highest level of attained education, Townsend deprivation index and alcohol intake status (Model 2), further adjusted for first-degree family history, cancer screening attendance, and time since screening attendance for the respective cancer.

Table 3. The association between the EAT-Lancet reference diet score and incident major cardiovascular events

	Incident major cardiovascular events HR (95% CI)				Stroke HR (95% CI)				Ischemic stroke HR (95% CI)				Hemorrhagic stroke HR (95% CI)				Myocardial infarction HR (95% CI)			
EAT-Lancet reference diet score	Number of cases[a]	Model 1[b]	Model 2[c]	Model 3c[d]	Number of cases[a]	Model 1[b]	Model 2[c]	Model 3c[d]	Number of cases[a]	Model 1[b]	Model 2[c]	Model 3c[d]	Number of cases[a]	Model 1[b]	Model 2[c]	Model 3c[d]	Number of cases[a]	Model 1[b]	Model 2[c]	Model 3c[d]
Continuous, 0-11	7,530	0.99 (0.98–1.01)	1.00 (0.98–1.01)	0.97 (0.96–0.98)	2,901	0.99 (0.97–1.01)	0.99 (0.97–1.02)	0.99 (0.97–1.02)	2,049	0.99 (0.96–1.02)	1.00 (0.97–1.03)	1.00 (0.97–1.03)	414	1.02 (0.95–1.08)	1.02 (0.95–1.08)	1.02 (0.95–1.08)	4,670	0.99 (0.97–1.01)	1.00 (0.99–1.02)	1.00 (0.98–1.02)
Low adherence, 0-4	3,794	Ref.	Ref.	Ref.	1,430	Ref.	Ref.	Ref.	1,000	Ref.	Ref.	Ref.	198	Ref.	Ref.	Ref.	2,384	Ref.	Ref.	Ref.
Moderate adherence, 5-7	3,383	0.97 (0.93–1.02)	0.98 (0.94–1.03)	0.92 (0.88–0.97)	1,336	0.99 (0.92–1.07)	1.00 (0.93–1.08)	1.00 (0.93–1.08)	954	1.01 (0.93–1.11)	1.02 (0.94–1.12)	1.02 (0.94–1.12)	194	1.03 (0.84–1.26)	1.04 (0.85–1.27)	1.04 (0.85–1.27)	2,067	0.96 (0.90–1.02)	0.97 (0.91–1.03)	0.97 (0.91–1.02)
High adherence, 8-11	353	1.07 (0.96–1.19)	1.09 (0.98–1.21)	0.94 (0.84–1.05)	135	1.02 (0.85–1.22)	1.04 (0.87–1.24)	1.03 (0.87–1.23)	95	1.02 (0.83–1.27)	1.04 (0.84–1.29)	1.04 (0.84–1.28)	22	1.17 (0.75–1.82)	1.18 (0.76–1.84)	1.18 (0.76–1.83)	219	1.10 (0.95–1.26)	1.12 (0.98–1.29)	1.11 (0.97–1.28)

Abbreviations: CI: confidence interval; HR: hazard ratio.

[a]A small number of participants was diagnosed with both stroke and myocardial infraction on the same day, thus the number of cases of the individual major cardiovascular events does not add up to the total. Similarly, a small number of participants was diagnosed with both main stroke subtypes on the same day.

[b]Model based on age, sex, and region.

[c]Model based on age, sex, and region plus further adjustment for smoking status, body mass index, physical activity, highest level of attained education, Townsend deprivation index and alcohol intake status.

[d]Model based on age, sex, region, smoking status, body mass index, physical activity, highest level of attained education, Townsend deprivation index and alcohol intake status (Model 2), further adjusted for first-degree family history for heart disease and stroke.

Table 4. The association between the EAT-Lancet reference diet score and all-cause mortality

		HR (95% CI)	
EAT-Lancet reference diet score	Number of cases	Model 1 [a]	Model 2 [b]
Continuous, 0–11	34,438	0.98 (0.97–0.99)	0.98 (0.98–0.99)
Low adherence, 0–4	17,178	Ref.	Ref.
Moderate adherence, 5–7	15,817	0.96 (0.94–0.98)	0.97 (0.95–0.99)
High adherence, 8–11	1,443	0.88 (0.83–0.93)	0.90 (0.85–0.95)

Abbreviations: CI: confidence interval; HR: hazard ratio.
[a]Model based on age, sex, and region.
[b]Model based on age, sex, and region plus further adjustment for smoking status, body mass index, physical activity, highest level of attained education, Townsend deprivation index and alcohol intake status.

the differences in how the scores were constructed, the follow-up time, and the different outcomes, many of the existing studies have reported a modest inverse association between the EAT-Lancet reference diet and non-communicable diseases[17,19,21] or mortality.[21,22] While considerable debate surrounds the potential adoption of the EAT-Lancet reference diet on a wide scale, particularly due to concerns regarding its affordability[25,26] and nutrient sufficiency,[27] this theoretical diet was designed, according to the EAT-Lancet Commission, to not exceed selected planetary resources. The existing evidence thus supports that adoption of a diet consistent with the EAT-Lancet reference diet cutoffs could have a dual benefit, both for the planet but also for the prevention of non-communicable diseases.

Given the complex, dynamic relationships in food systems, it seems unlikely that one single action (the widespread adoption of the EAT-Lancet reference diet or any other isolated action) will be able to effectively address the multifaceted challenges faced by food systems. This is supported by the EAT-Lancet Commission as well, which outlined five strategies for immediate food system transformation in their report.[4] A wide range of targeted, interconnected, cost-effective efforts addressing factors like food production, food processing and distribution, campaigns, food labeling, and food pricing strategies[28] is needed to institute meaningful changes.

The strengths of this study included the prospective study design and the substantial number of confirmed incident cases, but it also has some limitations. Dietary information was only available at baseline for the full cohort and thus may not reflect the long-term lifestyle habits of the study participants. To overcome this, we stratified the study results by self-reported dietary changes. Another limitation is an important criticism received for the EAT-Lancet reference diet, suggesting that the proposed dietary intake does not take into account the dietary needs of many population groups that are potentially susceptible to dietary deficiencies (e.g., females of reproductive age, children). To overcome this, we rescaled the diet proposed by the EAT-Lancet committee to reflect a daily intake of 2,000 kilocalories, which we used for females in our study. While we acknowledge the potential dietary deficiencies of the proposed diet for various population groups (e.g., females of reproductive age), these shortcomings seem less pertinent for our project since the inherent age inclusion criteria in the UK Biobank (mainly 40–69 years old) meant that few pregnant women are included in the study population. Nevertheless, these participants were excluded from our analyses. The dietary data collected with the touchscreen questionnaire did not include all relevant information, so some components of the EAT-Lancet reference diet could not be operationalized in our study. Due to the lack of information on first-degree family history for all cancer types, the analyses were only adjusted for first-degree family history for cancer only when information was available (i.e., breast, colorectal, and prostate cancer). As in all observational studies, the possibility of residual confounding, or the possibility that the study population might be more health-conscious than the general population cannot be excluded.

In conclusion, this large population-based study provides evidence to support the adherence to the diet proposed by the EAT-Lancet expert commission not only for mitigation of the climate emergency, but also for reduction in non-communicable diseases and all-cause mortality. Additional studies are needed, especially from non-Western countries, to further assess the link between the EAT-Lancet reference diet and non-communicable diseases in diverse populations.

EXPERIMENTAL PROCEDURES

Resource availability

Lead contact

Requests for further information and resources should be directed to the lead contact, Sabine Rohrmann (sabine.rohrmann@uzh.ch).

Materials availability

No materials were used in this study.

Data and code availability

This work has been conducted using the UK Biobank Resource (application number 81738). The UK Biobank is an open access resource and bona fide researchers can apply to use the UK Biobank dataset by registering and applying at http://ukbiobank.ac.uk/register-apply/. The code of this analysis has been submitted to the UK Biobank (as per contract).

Study population

The UK Biobank cohort is a large, population-based prospective study. More than 500,000 participants, mainly aged 40–69 years old, were recruited throughout the UK from 2006 to 2010. Detailed information on the study design, methods, and rationale of the cohort has been previously reported.[29] The UK Biobank has ethical approval from the North West Multi-centre Research Ethics Committee. All participants provided informed consent. Briefly, participants provided medical, dietary, and lifestyle data via a Touchscreen questionnaire. Physical measurements were also taken, and participants provided blood and urine samples. Participants were followed using linkages to routinely available central registers where incident cancers, hospital admissions, and deaths were reported.

A flowchart of the study population can be seen in Figure S1. Participants were excluded from the analyses if they had a recorded prevalent cancer (excluding non-melanoma skin cancer) or major cardiovascular event (stroke, myocardial infarction) diagnosis at recruitment in the UK Biobank (based on linkage data), if they were pregnant or had unknown pregnancy status at the time of recruitment, or if they had missing information on the included dietary variables. In analyses with cancer as the outcome, participants whose sex did not match their genetic sex were excluded. Participants whose date of cancer or major cardiovascular event diagnosis coincided with their date of death were also excluded.

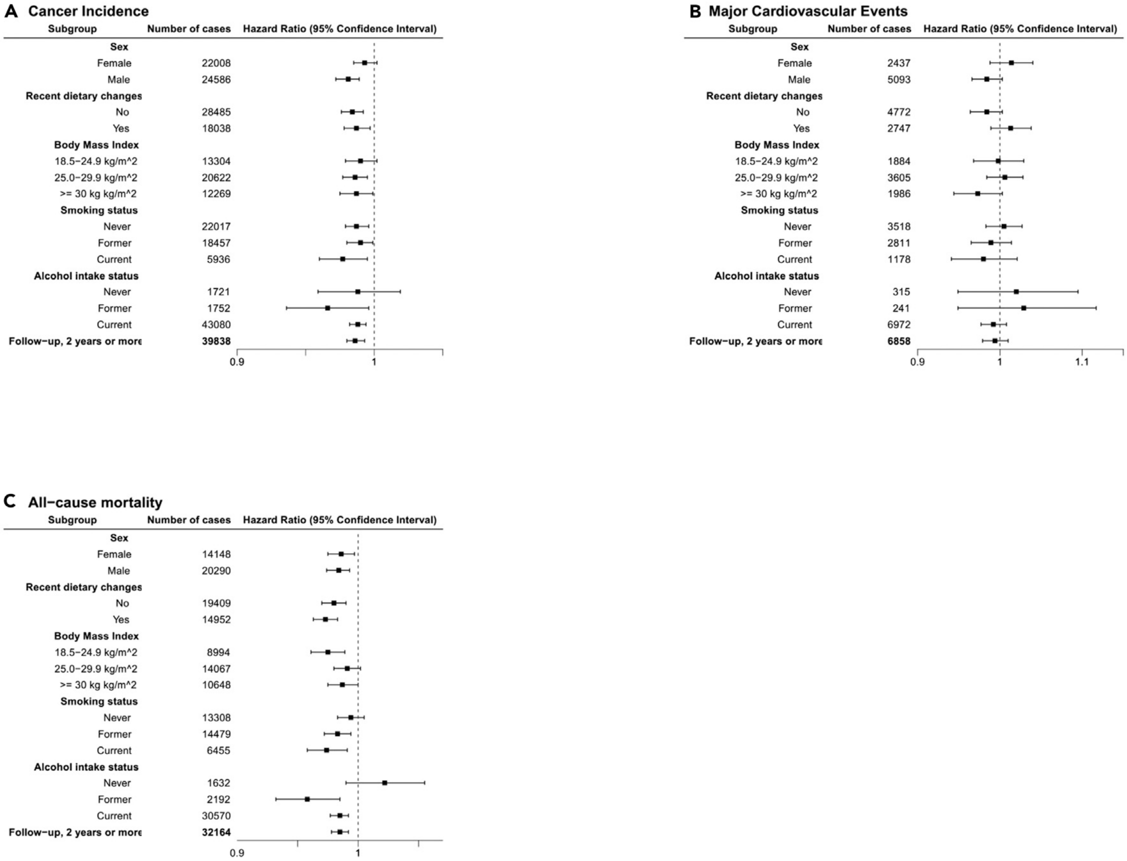

Figure 1. The association between the EAT-Lancet reference diet and other factors
(A) Cancer incidence, stratified by pre-selected lifestyle factors, (B) incident major cardiovascular events, stratified by pre-selected lifestyle factors, and (C) all-cause mortality, stratified by pre-selected lifestyle factors.

Dietary variables

Dietary information collected via the UK Biobank Touchscreen questionnaire was used to develop a score that reflects adherence to the EAT-Lancet reference diet. The Touchscreen questionnaire inquired about the consumption frequency over the past year (29 questions). The items assessed were cooked vegetables, salad/raw vegetables, fresh fruit, dried fruit, oily fish, other fish, processed meats, poultry, beef, lamb, pork, cheese, salt added to food, tea, and water. Additionally, questions on the type of milk and spread most commonly consumed, number of slices and type of bread most commonly consumed, number of bowls and type of breakfast cereal most commonly consumed, cups of coffee and type most commonly consumed were asked. Furthermore, the avoidance of specific food groups (eggs or foods containing eggs, dairy products, wheat products, sugar or foods/drinks containing sugar), the age when the participant last ate meat, the preferred temperature of hot drinks, changes in diet in the past 5 years, and variation in diet were also assessed.

Food items whose consumption was asked at a weekly level were transformed into daily consumption (in grams per day). Standard portion sizes reported for the United Kingdom[30] were used to transform consumption to the necessary level of daily consumption.

Adherence to the EAT-Lancet reference diet

Participants received a score based on whether each component of their diets (e.g., daily fruit intake; Table S1) was within the cutoffs of the EAT-Lancet reference diet. The components we were able to use were grains, vegetables, fruits, dairy foods, protein sources (i.e., beef, lamb, and pork, chicken and other poultry, eggs, fish), added fats (unsaturated oils, saturated oils), and added sugars. Due to lack of information in the UK Biobank Touchscreen questionnaire, we were not able to use the components on tuber or starchy vegetables, legumes, or nuts. Our scoring approach closely resembles the one described by Knuppel et al.[17] When the UK Biobank Touchscreen information did not sufficiently capture the food group, different scoring was used (Table S1).

The cutoffs for the EAT-Lancet diet are based on a diet of 2,500 kcal/day.[4] Since females have been known to consume less than males, the dietary intake for females was rescaled to reflect a diet of 2,000 kcal/day, rounded to the nearest whole number. For each component, participants received one point if their consumption was within the cutoff values. Zero points were awarded if the consumption was outside of the cutoff values. The sum of all components resulted in each participant's overall EAT-Lancet reference diet score. The overall score was constructed such that each component contributes equally to the EAT-Lancet reference diet score.

Due to the lack of information on the exact consumption of certain EAT-Lancet reference diet components in the UK Biobank Touchscreen questionnaire, the self-reported avoidance of these food groups was used (Table S1). Based on the question "Which of the following do you never eat?" with possible answers including eggs or foods containing eggs, dairy products, wheat products, sugar or foods/drinks containing sugar, participants received one point if they avoided these components. The score ranged from 0 to 11, with higher scores reflecting greater adherence to the EAT-Lancet reference diet. The score was additionally categorized into three groups: low adherence (0–4 points), moderate adherence (5–7 points), and high adherence (8–11 points).

Case ascertainment

Participants diagnosed with first primary incident cancer, based on cancer registry information, between recruitment and the latest date of complete information were considered as incident cancer cases. The latest dates of

complete information for cancer incidence varied among countries (February 29, 2020, for England and Wales; January 31, 2021, for Scotland). Cancer cases were coded according to the 10th Revision of the International Statistical Classification of Diseases, Injuries, and Causes of Death. Cases recorded as C00-C97, as well as D320, D321, D329, D330, D332, D333, D334, D339, D352, D420, D429, D430, D431, D432, D439, D443, D444, D445, excluding C44, were considered as incident cancer cases.

Participants diagnosed with first primary incident major cardiovascular events, based on hospital admissions, between recruitment and the latest date of complete information were considered as incident cardiovascular event cases. The latest dates of complete information for hospital admissions varied among countries (September 30, 2021, for England; March 31, 2016, for Wales; July 31, 2021, for Scotland). Cases were coded according to the 10th Revision of the International Statistical Classification of Diseases, Injuries, and Causes of Death. Cases recorded as I60, I61, I63, I64, I21, I22, I23, I241, and I252 were considered major cardiovascular event cases.

Participants who were reported to be dead, based on central registers, between recruitment and the latest date of complete information (September 30, 2021, for England and Wales; October 31, 2021, for Scotland) were considered as mortality cases.

Adjusting variables

Upon recruitment, medical, dietary, anthropometric, and lifestyle data were collected, including information on alcohol use, smoking status, physical activity, education, reproductive history, and previous illnesses. Body size measurements were also taken. The baseline questionnaire also asked participants to estimate the number of days in a typical week they engage in moderate and vigorous physical activity, as well as the typical duration of these activities (in minutes; separate questions for moderate and vigorous activities) on a typical day.

Statistical analyses

Categorical variables were presented by percentages and continuous variables by arithmetic means and standard deviations (SDs) for descriptive purposes.

Cox proportional hazards regression models were used to assess the association of the EAT-Lancet reference diet score with incident cancer (overall and for selected cancer sites), incident major cardiovascular events, and all-cause mortality. Entry time was defined as a participant's age at study recruitment and exit time as a participant's age at the first incident cancer diagnosis, incident cardiovascular event or death (depending on the analytical subsample), loss to follow-up, or end of follow-up, whichever came first.

The EAT-Lancet reference diet score was assessed as continuous and as categorical variables. Categories, as mentioned above, were created to reflect low, moderate, or high adherence to the EAT-Lancet reference diet.

Confounding adjustment followed a tiered approach. Model 1 was stratified for age (5-year intervals), region at study assessment (10 regions), and sex. Model 2 was further adjusted for education level, Townsend deprivation index (quintiles; as an indicator for socio-economic status), smoking status (never, former, current, prefer not to answer/missing), BMI categories ($\leq$18.5 kg/m^2, 18.5–24.9 kg/m^2, 25.0–29.9 kg/m^2, $\geq$30 kg/m^2, missing; according to the World Health Organization classification[31]), physical activity (<75 min per week, 75–150 min per week, >150 min per week, prefer not to answer/missing) and alcohol intake status (never, former, current drinker, prefer not to answer/missing). Model 2 is considered the main model in our analyses. In analyses with breast cancer as the outcome, reproductive factors (i.e., parity, age at menopause, menopausal status, use of oral contraceptives, or menopausal hormone therapy) were included in an additional model (Model 3a). Analyses with breast, colorectal, and prostate cancers as outcomes were also adjusted for first-degree family history for each respective cancer, previous cancer screening attendance (breast, colorectal, prostate-specific antigen test), and time since the last screening in an additional model (Model 3b). Analyses with major cardiovascular events as the outcome were additionally adjusted for first-degree family history of heart disease and stroke (Model 3c).

Pre-planned stratified analyses by potential effect modifiers were conducted including by sex, BMI, smoking status, alcohol intake status, and self-reported changes in diet in the past 5 years. Confounding adjustment in these analyses followed Model 2, as described above. When one of the adjustment variables was used as a stratifying variable, it was not included in the model. Sensitivity analyses were conducted restricting the analyses to participants with at least 2 years of follow-up time. Analyses were conducted using Stata version 13 (StataCorp, Texas). All statistical tests were two-sided, and p values <0.05 were considered statistically significant.

SUPPLEMENTAL INFORMATION

Supplemental information can be found online at https://doi.org/10.1016/j.oneear.2023.11.002.

ACKNOWLEDGMENTS

This work was conducted using the UK Biobank Resource under Application Number 81738. Funding for grant IIG_FULL_2021_012 was obtained from the World Cancer Research Fund (WCRF UK), as part of the World Cancer Research Fund International grant program. The funder had no role in study design, data analysis, decision to publish, or preparation of the manuscript. Dr. Papier was supported by the Wellcome Trust, Our Planet Our Health (Livestock, Environment and People—LEAP) [grant number 205212/Z/16/Z] and Cancer Research UK [grant number C570/A16491 and C16077/A29186]. At the time of publication, Dr. Karavasiloglou is an EFSA staff member. At the time of preparation of this work, Dr. Karavasiloglou was affiliated with the University of Zurich and the University Hospital Zurich. Where authors are identified as personnel of the European Food Safety Authority, the authors alone are responsible for the views expressed in this article and they do not necessarily represent the decisions, policy or views of the European Food Safety Authority.

AUTHOR CONTRIBUTIONS

Conception and design: N.K., A.T., G.P., A.K., K.P., A.C., T.K., S.R. Data acquisition: N.K., S.R. Analyzing the data: N.K., A.T., T.K., S.R. Interpretation of the data: N.K., A.T., G.P., A.C., T.K., S.R. Verifying the data: A.T., A.K., K.P. Drafting the manuscript: N.K. Critically revising the manuscript: N.K., A.T., G.P., A.K., K.P., A.C., T.K., S.R. All authors read and approved the final manuscript.

DECLARATION OF INTERESTS

The authors declare no competing interests.

Received: March 19, 2023
Revised: June 30, 2023
Accepted: November 1, 2023
Published: November 21, 2023

REFERENCES

1. Vermeulen, S.J., Campbell, B.M., and Ingram, J.S. (2012). Climate Change and Food Systems. Annu. Rev. Environ. Resour. *37*, 195–222.
2. Burlingame, B., and Dernini, S. (2012). Sustainable Diets and Biodiversity Directions and Solutions for Policy, Research and Action.
3. Willett, W., Rockström, J., Loken, B., Springmann, M., Lang, T., Vermeulen, S., Garnett, T., Tilman, D., DeClerck, F., Wood, A., et al. (2019). Food in the Anthropocene: the EAT–Lancet Commission on healthy diets from sustainable food systems. Lancet *393*, 447–492.
4. EAT-Lancet Commission (2019). Summary Report of the EAT-Lancet Commission (Food Planet Health. Healthy Diets From Sustainable Food Systems).
5. GBD 2017 Diet Collaborators (2019). Health effects of dietary risks in 195 countries, 1990-2017: a systematic analysis for the Global Burden of Disease Study 2017. Lancet Lond. Engl. *393*, 1958–1972.
6. Bechthold, A., Boeing, H., Schwedhelm, C., Hoffmann, G., Knüppel, S., Iqbal, K., De Henauw, S., Michels, N., Devleesschauwer, B., Schlesinger, S., and Schwingshackl, L. (2019). Food groups and risk of coronary heart disease, stroke and heart failure: A systematic review

and dose-response meta-analysis of prospective studies. Crit. Rev. Food Sci. Nutr. *59*, 1071–1090.

7. Schwedhelm, C., Boeing, H., Hoffmann, G., Aleksandrova, K., and Schwingshackl, L. (2016). Effect of diet on mortality and cancer recurrence among cancer survivors: a systematic review and meta-analysis of cohort studies. Nutr. Rev. *74*, 737–748.
8. Vineis, P., and Wild, C.P. (2014). Global cancer patterns: causes and prevention. Lancet Lond. Engl. *383*, 549–557.
9. Schüz, J., Espina, C., Villain, P., Herrero, R., Leon, M.E., Minozzi, S., Romieu, I., Segnan, N., Wardle, J., Wiseman, M., et al. (2015). European Code against Cancer 4th Edition: 12 ways to reduce your cancer risk. Cancer Epidemiol. *39* (*Suppl 1*), S1–S10.
10. Behrens, G., Gredner, T., Stock, C., Leitzmann, M.F., Brenner, H., and Mons, U. (2018). Cancers Due to Excess Weight, Low Physical Activity, and Unhealthy Diet. Dtsch. Arztebl. Int. *115*, 578–585.
11. World Cancer Research Fund/American Institute for Cancer Research (2018). Diet, Nutrition, Physical Activity and Cancer: a Global Perspective. Continuous Update Project Expert Report. dietandcancerreport.org.
12. Schwingshackl, L., Schwedhelm, C., Hoffmann, G., Lampousi, A.-M., Knüppel, S., Iqbal, K., Bechthold, A., Schlesinger, S., and Boeing, H. (2017). Food groups and risk of all-cause mortality: a systematic review and meta-analysis of prospective studies. Am. J. Clin. Nutr. *105*, 1462–1473.
13. Islami, F., Goding Sauer, A., Miller, K.D., Siegel, R.L., Fedewa, S.A., Jacobs, E.J., McCullough, M.L., Patel, A.V., Ma, J., Soerjomataram, I., et al. (2018). Proportion and number of cancer cases and deaths attributable to potentially modifiable risk factors in the United States. CA. Cancer J. Clin. *68*, 31–54.
14. Petermann-Rocha, F., Ho, F.K., Foster, H., Boopor, J., Parra-Soto, S., Gray, S.R., Mathers, J.C., Celis-Morales, C., and Pell, J.P. (2021). Nonlinear Associations Between Cumulative Dietary Risk Factors and Cardiovascular Diseases, Cancer, and All-Cause Mortality: A Prospective Cohort Study From UK Biobank. Mayo Clin. Proc. *96*, 2418–2431.
15. Schwingshackl, L., Schwedhelm, C., Hoffmann, G., Knüppel, S., Laure Preterre, A., Iqbal, K., Bechthold, A., De Henauw, S., Michels, N., Devleesschauwer, B., et al. (2018). Food groups and risk of colorectal cancer. Int. J. Cancer *142*, 1748–1758.
16. Schwingshackl, L., Hoffmann, G., Lampousi, A.-M., Knüppel, S., Iqbal, K., Schwedhelm, C., Bechthold, A., Schlesinger, S., and Boeing, H. (2017). Food groups and risk of type 2 diabetes mellitus: a systematic review and meta-analysis of prospective studies. Eur. J. Epidemiol. *32*, 363–375.
17. Knuppel, A., Papier, K., Key, T.J., and Travis, R.C. (2019). EAT-Lancet score and major health outcomes: the EPIC-Oxford study. Lancet *394*, 213–214.
18. Berthy, F., Brunin, J., Allès, B., Fézeu, L.K., Touvier, M., Hercberg, S., Galan, P., Pointereau, P., Lairon, D., Baudry, J., and Kesse-Guyot, E. (2022). Association Between Adherence to the EAT-Lancet Diet and Risk of Cancer and Cardiovascular Outcomes in the Prospective NutriNet-Santé Cohort. Am. J. Clin. Nutr. *116*, 980–991.
19. Ibsen, D.B., Christiansen, A.H., Olsen, A., Tjønneland, A., Overvad, K., Wolk, A., Mortensen, J.K., and Dahm, C.C. (2022). Adherence to the EAT-Lancet Diet and Risk of Stroke and Stroke Subtypes: A Cohort Study. Stroke *53*, 154–163.
20. Xu, C., Cao, Z., Yang, H., Hou, Y., Wang, X., and Wang, Y. (2021). Association Between the EAT-Lancet Diet Pattern and Risk of Type 2 Diabetes: A Prospective Cohort Study. Front. Nutr. *8*, 784018.
21. Colizzi, C., Harbers, M.C., Vellinga, R.E., Verschuren, W.M.M., Boer, J.M.A., Biesbroek, S., Temme, E.H.M., and van der Schouw, Y.T. (2023). Adherence to the EAT-Lancet Healthy Reference Diet in relation to Coronary Heart Disease, All-Cause Mortality Risk and Environmental Impact: Results from the EPIC-NL Cohort. J. Am. Heart Assoc. *12*, e026318.
22. Stubbendorff, A., Sonestedt, E., Ramne, S., Drake, I., Hallström, E., and Ericson, U. (2022). Development of an EAT-Lancet index and its relation to mortality in a Swedish population. Am. J. Clin. Nutr. *115*, 705–716.
23. Zhang, S., Dukuzimana, J., Stubbendorff, A., Ericson, U., Borné, Y., and Sonestedt, E. (2023). Adherence to the EAT-Lancet diet and risk of coronary events in the Malmö Diet and Cancer cohort study. Am. J. Clin. Nutr. *117*, 903–909.
24. Zhang, S., Stubbendorff, A., Olsson, K., Ericson, U., Niu, K., Qi, L., Borné, Y., and Sonestedt, E. (2023). Adherence to the EAT-Lancet diet, genetic susceptibility, and risk of type 2 diabetes in Swedish adults. Metabolism *141*, 155401.
25. Goulding, T., Lindberg, R., and Russell, C.G. (2020). The affordability of a healthy and sustainable diet: an Australian case study. Nutr. J. *19*, 109.
26. Hirvonen, K., Bai, Y., Headey, D., and Masters, W.A. (2020). Affordability of the EAT-Lancet reference diet: a global analysis. Lancet Global Health *8*, e59–e66.
27. Beal, T., Ortenzi, F., and Fanzo, J. (2023). Estimated micronutrient shortfalls of the EAT-Lancet planetary health diet. Lancet Planet. Health *7*, e233–e237.
28. Webb, P., Benton, T.G., Beddington, J., Flynn, D., Kelly, N.M., and Thomas, S.M. (2020). The urgency of food system transformation is now irrefutable. Nat. Food *1*, 584–585.
29. Sudlow, C., Gallacher, J., Allen, N., Beral, V., Burton, P., Danesh, J., Downey, P., Elliott, P., Green, J., Landray, M., et al. (2015). UK biobank: an open access resource for identifying the causes of a wide range of complex diseases of middle and old age. PLoS Med. *12*, e1001779.
30. (2022). British Dietetic Association Portion sizes: Food Fact Sheet.
31. World Health Organization (2000). Obesity: Preventing and Managing the Global Epidemic (World Health Organization).

One Earth, Volume *6*

Supplemental information

Adherence to the EAT-Lancet reference diet is associated with a reduced risk of incident cancer and all-cause mortality in UK adults

Nena Karavasiloglou, Alysha S. Thompson, Giulia Pestoni, Anika Knuppel, Keren Papier, Aedín Cassidy, Tilman Kühn, and Sabine Rohrmann

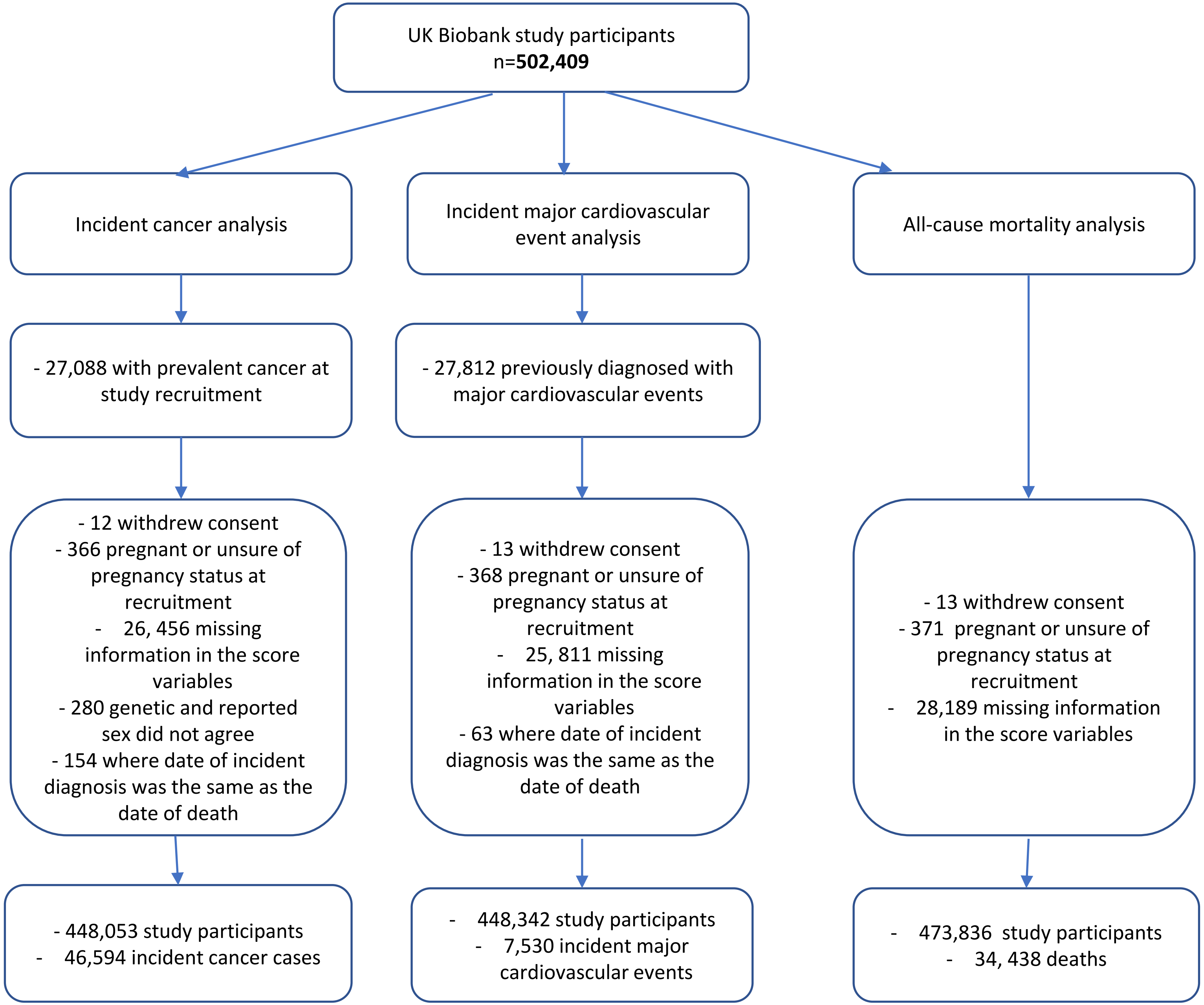

Figure S1. Flowchart of study population

Table S1. Operationalization of the components of the EAT-Lancet reference diet using the UK Biobank Touchscreen questionnaire [a,b]

EAT-Lancet reference diet component	Food groups in the UK Biobank	Cut-offs of daily dietary intake (males/females)
Grains (incl. rice, wheat, corn, and other)	Partial component, estimated based on bread intake and cereal intake and on the question "Which of the following do you never eat?", with answer wheat products	Sex-specific cohort-based median of consumers.<br>> 91g/ 70g (median): 0<br>≤ median: 1<br>Participants reporting avoidance of wheat products and reported no consumption of cereals received the one point.
Vegetables	Cooked vegetable and salad / raw vegetable intake	200-600g/160-480g
Fruits	Fresh fruit and dried fruit intake	100-300g/80-240g
Dairy foods (incl. whole milk or equivalents)	Partial component, estimated based on cheese intake and on the question "Which of the following do you never eat?", with possible answer dairy products	Sex-specific cohort-based median of consumers.<br>> 13g/ 13g (median): 0<br>≤ median: 1<br><br>Participants reporting avoidance of dairy received one point
Protein sources		
Beef, lamb, and pork	Beef, lamb/mutton intake and pork intake	≤28g/22g
Chicken and other poultry	Poultry	≤58g/46g
Eggs	Binary outcome, based on the question "Which of the following do you never eat?", with possible answer eggs	Participants avoiding eggs received one point
Fish	Oily fish and non-oily fish intake	≤100g/80g
Added fats		
Unsaturated oils	Binary outcome, based on spread type and non-butter spread type details	Those not using spreads or using Benecol received the full point. The rest and those using hard margarine received one point
Saturated oils	Binary outcome, based on spread type and non-butter spread type details	Those not using spreads or using Benecol received the full point. The rest and those using hard margarine received one point
Added sugars (incl. all sugars)	Binary outcome, based on the question "Which of the following do you never eat?", with possible answer sugar or foods/ drinks containing sugar	Participants avoiding sugar received one point

[a] The EAT-Lancet reference diet cut-offs are based on the report of the EAT-Lancet Commission, to reflect a diet of 2,500 kcal/day. (Summary Report of the EAT-Lancet Commission. Food Planet Health. Healthy Diets From Sustainable Food Systems, 2019). Our score is based, to the extend possible, on the work by Knuppel et al. 2019. Consumption within each component's cut-offs results in one point being awarded, otherwise, zero points are awarded. When the information in the UK Biobank Touchscreen questionnaire was not detailed enough to allow us to follow the score by Knuppel et al. 2019, the sex-specific cohort medians were used as a cut-off.

[b] The dietary intake for females was rescaled to reflect a diet of 2,000 kcal/day, rounded to the nearest whole number. Due to the selected food groups in the Touchscreen questionnaire, it was not possible to estimate the consumption of tuber or starchy vegetables, legumes, and nuts, and include them in the score.

Note S1. STROBE Statement for cohort studies

	Ite m No	Recommendation	Page No
**Title and abstract**	1	(*a*) Indicate the study's design with a commonly used term in the title or the abstract	2
		(*b*) Provide in the abstract an informative and balanced summary of what was done and what was found	2
**Introduction**			
Background/rationale	2	Explain the scientific background and rationale for the investigation being reported	3-4
Objectives	3	State specific objectives, including any prespecified hypotheses	4
**Methods**			
Study design	4	Present key elements of study design early in the paper	4-8
Setting	5	Describe the setting, locations, and relevant dates, including periods of recruitment, exposure, follow-up, and data collection	4-8
Participants	6	(*a*) Give the eligibility criteria, and the sources and methods of selection of participants. Describe methods of follow-up	4, 6
		(*b*) For matched studies, give matching criteria and number of exposed and unexposed	
Variables	7	Clearly define all outcomes, exposures, predictors, potential confounders, and effect modifiers. Give diagnostic criteria, if applicable	4-8
Data sources/ measurement	8*	For each variable of interest, give sources of data and details of methods of assessment (measurement). Describe comparability of assessment methods if there is more than one group	4-8
Bias	9	Describe any efforts to address potential sources of bias	8
Study size	10	Explain how the study size was arrived at	4, Fig1
Quantitative variables	11	Explain how quantitative variables were handled in the analyses. If applicable, describe which groupings were chosen and why	5-8
Statistical methods	12	(*a*) Describe all statistical methods, including those used to control for confounding	7-8
		(*b*) Describe any methods used to examine subgroups and interactions	8
		(*c*) Explain how missing data were addressed	7-8
		(*d*) If applicable, explain how loss to follow-up was addressed	
		(*e*) Describe any sensitivity analyses	8
**Results**			
Participants	13*	(a) Report numbers of individuals at each stage of study—eg numbers potentially eligible, examined for eligibility, confirmed eligible, included in the study, completing follow-up, and analysed	Fig1
		(b) Give reasons for non-participation at each stage	Fig1
		(c) Consider use of a flow diagram	Fig1
Descriptive data	14*	(a) Give characteristics of study participants (eg demographic, clinical, social) and information on exposures and potential confounders	Table 2
		(b) Indicate number of participants with missing data for each variable of interest	Table 2
		(c) Summarise follow-up time (eg, average and total amount)	8

Outcome data	15*	Report numbers of outcome events or summary measures over time	Tables 3-5
Main results	16	(*a*) Give unadjusted estimates and, if applicable, confounder-adjusted estimates and their precision (eg, 95% confidence interval). Make clear which confounders were adjusted for and why they were included	Tables 3-5
		(*b*) Report category boundaries when continuous variables were categorized	Tables 3-5
		(*c*) If relevant, consider translating estimates of relative risk into absolute risk for a meaningful time period	n/a
Other analyses	17	Report other analyses done—eg analyses of subgroups and interactions, and sensitivity analyses	8, Fig 2-4
**Discussion**			
Key results	18	Summarise key results with reference to study objectives	9-10
Limitations	19	Discuss limitations of the study, taking into account sources of potential bias or imprecision. Discuss both direction and magnitude of any potential bias	10-11
Interpretation	20	Give a cautious overall interpretation of results considering objectives, limitations, multiplicity of analyses, results from similar studies, and other relevant evidence	11
Generalisability	21	Discuss the generalisability (external validity) of the study results	11
**Other information**			
Funding	22	Give the source of funding and the role of the funders for the present study and, if applicable, for the original study on which the present article is based	12

*Give information separately for exposed and unexposed groups.

Note: An Explanation and Elaboration article discusses each checklist item and gives methodological background and published examples of transparent reporting. The STROBE checklist is best used in conjunction with this article (freely available on the Web sites of PLoS Medicine at http://www.plosmedicine.org/, Annals of Internal Medicine at http://www.annals.org/, and Epidemiology at http://www.epidem.com/). Information on the STROBE Initiative is available at http://www.strobe-statement.org.

Supplemental reference list

EAT-Lancet Commission. Summary Report of the EAT-Lancet Commission. Food Planet Health. Healthy Diets From Sustainable Food Systems. 2019.

Knuppel A, Papier K, Key TJ, Travis RC. EAT-Lancet score and major health outcomes: the EPIC-Oxford study. *The Lancet* 2019; **394**: 213–4.
